# Prevaccination Prevalence of Type-Specific Human Papillomavirus Infection by Grade of Cervical Cytology in Estonia

**DOI:** 10.1001/jamanetworkopen.2022.54075

**Published:** 2023-02-06

**Authors:** Anneli Uusküla, Marek Oja, Sirli Tamm, Anna Tisler, Made Laanpere, Lee Padrik, Mari Nygard, Sulev Reisberg, Jaak Vilo, Raivo Kolde

**Affiliations:** 1 Department of Family Medicine and Public Health, University of Tartu, Tartu, Estonia; 2 Institute of Computer Science, University of Tartu, Tartu, Estonia; 3 STACC, Tartu, Estonia; 4 Department of Obstetrics and Gynaecology, Institute of Clinical Medicine, University of Tartu; Tartu University Hospital Women’s Clinic, Tartu, Estonia; 5 Department of Research, Cancer Registry of Norway, Oslo, Norway; 6 Quretec, Tartu, Estonia

## Abstract

**Importance:**

Large-scale data on type-specific human papillomavirus (HPV) prevalence and disease burden worldwide are needed to guide cervical cancer prevention efforts. Promoting the research and application of health care big data has become a key factor in modern medical research.

**Objective:**

To examine the prevaccination prevalence of high-risk HPV (hrHPV) and type distribution by cervical cytology grade in Estonia.

**Design, Setting, and Participants:**

This cross-sectional study used text mining and the linking of data from electronic health records and health care claims to examine type-specific hrHPV positivity in Estonia from 2012 to 2019. Participants were women aged at least 18 years. Statistical analysis was performed from September 2021 to August 2022.

**Main Outcomes and Measures:**

Type-specific hrHPV positivity rate by cervical cytological grade.

**Results:**

A total of 11 017 cases of cervical cytology complemented with data on hrHPV testing results between 2012 and 2019 from 66 451 women aged at least 18 years (mean [SD] age, 48.1 [21.0] years) were included. The most common hrHPV types were HPV16, 18, 31, 33, 51 and 52, which accounted for 73.8% of all hrHPV types detected. There was a marked decline in the positivity rate of hrHPV infection with increasing age, but the proportion did not vary significantly based on HPV type. Implementation of nonavalent prophylactic vaccination was estimated to reduce the number of women with high-grade cytology by 50.5% (95% CI, 47.4%-53.6%) and the number with low-grade cytology by 27.8% (95% CI, 26.3%-29.3%), giving an overall estimated reduction of 33.1% (95% CI, 31.7%-34.5%) in the number of women with precancerous cervical cytology findings.

**Conclusions and Relevance:**

In this cross-sectional study, text mining and natural language processing techniques allowed the detection of precursors to cervical cancer based on data stored by the nationwide health system. These findings contribute to the literature on type-specific HPV distribution by cervical cytology grade and document that α-9 phylogenetic group HPV types 16, 31, 33, 52 and α-7 phylogenetic group HPV 18 are the most frequently detected in normal-to-high-grade precancerous lesions in Estonia.

## Key Points

**Question:**

What is the estimated prevaccination prevalence and type distribution of high-risk human papillomavirus (hrHPV) by cytological cervical precancerous grade in Estonia?

**Findings:**

In this cross-sectional study leveraging text mining on nationwide health data of 66 451 women in Estonia, the 6 most common hrHPV (16, 18, 31, 33, 51, 52) accounted for 74% of all hrHPV types detected. Nonavalent prophylactic vaccination was estimated to prevent 50.5% of high-grade cytology cases and 27.8% of low-grade cytology cases.

**Meaning:**

These findings suggest that text mining and linking of health care data may be useful for informing preventive efforts for cervical cancer.

## Teaser

This cross-sectional study examines the prevaccination prevalence of high-risk human papillomavirus (HPV) and type distribution by cervical cytology grade in Estonia.

## Introduction

Infection with high-risk human papillomavirus (hrHPV) is one of the definitive events linked to the complex process of cervical carcinogenesis.^1^ The knowledge of HPV genotype distribution in cervical neoplasia is valuable for predicting the effectiveness of current HPV vaccines on cancer prevention. HPV infections are common among young women, with most infections spontaneously clearing within 1 to 2 years.^2^ Persistent infection with hrHPV is considered essential for the development of cervical cancer.^3^ Evidence on the occurrence of hrHPV infection and related diseases represents a crucial topic for predicting and evaluating antiviral prophylactic vaccines and informing the design of therapeutic vaccines.

Previous reports have documented significant regional differences in hrHPV prevalence worldwide (including in women with normal cytology and invasive cancer).^4,5,6^ Prevalence of HPV infection and type-specific distribution vary greatly between nations^7^ and between grades of cervical cytology.^8,9,10,11,12,13^ Data on the prevalence and distribution of HPV types by cervical cytology grade from regions in Europe characterized by high cervical cancer incidence and mortality are scarce.^6,7,14^

Various types of medical data (electronic health records, billing data, registries) are generated at a high speed and the growth in research involving data mining (DM) and artificial intelligence (AI) in health care has been exponential. A recent study assessing translatability (from algorithms that perform well on standardized data sets to operational systems demonstrated an improvement in clinical outcomes^15^) of AI applications brought about electronic health record–based research among those with the highest promise for health improvement.^16^

We applied data mining methodology on data from nationwide and a population-based electronic health record (EHR) system to examine the prevaccination prevalence of hrHPV and the distribution of HPV types by cervical cytology grade in the Estonian female population. In addition, we evaluated the potential effects of HPV vaccines in Estonia.

## Methods

### Ethics

This cross-sectional study was approved by the Research Ethics Committee of the University of Tartu and the Estonian Committee on Bioethics and Human Research and conducted as part of Activity 1: Support for Strategic R&D Activities—machine learning and AI-powered public service delivery—within the ongoing project Strengthening of Sectoral Research and Development. The requirement for informed consent was waived due to minimal risk in retrospective studies. This study followed the Strengthening the Reporting of Observational Studies in Epidemiology (STROBE) reporting guideline.

### Setting

In Estonia, the estimated age-standardized incidence of cervical cancer was 14.4 per 100 000 women from 2014 to 2018.^17^ This is almost double the incidence estimated in Western Europe (6.8 per 100 000), North America (6.4 per 100 000), and Australia (6.0 per 100 000).^14^ Over the past decades, the distribution of cervical cancer at the time of diagnosis has shifted toward the later stages.^18^ An organized effort at cervical cancer screening using cytology-based Papanicolaou testing every 5 years was introduced in 2006 in Estonia, targeting women aged 30 to 55 years. Cervical cancer screening attendance has been low and did not exceed 50%.^18^ Cytological cell samples were categorized according to the Bethesda system criteria as follows: negative for intraepithelial lesion or malignancy (NILM); atypical squamous cells of undetermined significance (ASCUS); atypical glandular cells not otherwise specified (AGC-NOS); low-grade squamous intraepithelial lesions (LSIL); atypical squamous cells cannot exclude HSIL (ASC-H); high-grade squamous intraepithelial lesions (HSIL); atypical glandular cells favor neoplasia (AGC-FN); and cervical cancer.^19^ Since 2014, hrHPV detection was formally recommended as a triage test.^20^ In addition, over the past decade, hrHPV testing has also been used for other indications (eg, clinical case diagnosis, test of cure).

In 2021, in Estonia, a 5-yearly primary HPV screening targeting women aged 30 to 65 years was implemented. HPV vaccination in Estonia began in 2018 for girls aged 12 to 14 years using the nonavalent vaccine.

HPV testing was done in ISO-accredited laboratories using Luminex xMAP (Luminex Corporation; detecting HPV genotypes 16, 18, 31, 33, 35, 39, 45, 51, 52, 53, 56, 58, 59, 66, 68, 82); and Cobas 4800 HPV test (Roche Molecular Diagnostics; HPV genotypes 16, 18 and pooled aggregate 31/33/35/39/45/51/52/56/58/59/66/68).

### Study Design

This cross-sectional study utilized data from Estonia’s nationwide and population-based electronic health data collection. These data were used to examine the prevaccination prevalence of hrHPV and the distribution of HPV type by cervical cytology grade in the Estonian female population.

### Data Source

We used data from an existing population-based cohort study that used a simple random sampling procedure to incorporate 10% of the Estonian population. From this source, cohort data on all women aged 18 years or older (n = 66451) were analyzed in this study. For each woman, all EHRs from the Health and Welfare Information Systems Centre^21^ and claims from Estonian Health Insurance Fund (EHIF)^22^ were obtained.

Data from EHR documents (*International Statistical Classification of Diseases and Related Health Problems, Tenth Revision [ICD-10]* diagnoses, HPV testing and cytology exams with results and dates, date of birth) and EHIF insurance claims (*ICD-10* diagnoses, Papanicolaou-testing episode dates, date of birth) were used for the period January 1, 2012, to December 31, 2019. The EHR contains a mix of semistructured and completely unstructured data. Text mining and natural language processing techniques were applied to extract Papanicolaou and HPV test results from the EHR. For cytology examination results, we first narrowed the scope to search only for gynecological cytology test results (using either keyword referring to gynecological procedures or locally used procedure and observation codes). Part of the results was free-text written by the pathologist.

In this case, the results were extracted by searching for specific concepts (eg, NILM) or codes (eg, SNOMED or SNOMED-CT codes, such as M00120 for NILM) or keywords and their modifications (eg, “pap II norm” for NILM) (See eTable 2 in Supplement 1 for concept sets specification.) It was possible that from one Papanicolaou test multiple results were extracted (eg, NILM and ASCUS). Then, the most severe condition was used in the analysis. The HPV testing results were searched from laboratory measurements. The HPV test was identified using LOINC (logical observation identifiers names and codes) code if available or by looking at measurement name pointing to HPV test related to the cervix. We excluded HPV tests not related to the cervix. At first, we detected whether the test result was positive or negative using keywords. If there was additional information about specific HPV types, it was extracted. Sometimes the particular HPV type was inferred from the test name (eg, if the person was tested only for HPV 16 and the result was positive, we concluded the HPV type to be 16). If information about a specific HPV type was missing and not derivable from the analysis name, the test was defined as hrHPV positive (with unspecified type). The linkage of the EHR and EHIF databases is virtually complete due to the use of unique personal identification codes, which are assigned to all Estonian residents at birth or at the time of immigration. Data were harmonized, linked, and mapped to the OHDSI (The Observational Health Data Sciences and Informatics)/OMOP (The Observational Medical Outcomes Partnership) common data model version 5.3.^23^

### High-risk HPV Infection

We describe HPV infections as identified on ad hoc testing. The episodes of HPV testing were based on the identification of HPV DNA test results and the corresponding dates. In addition to individual HPV type, the following HPV subgroups were examined: (1) HPV 16, 18; (2) HPV 31, 33, 45, 52 and 58 (the 5 additional types in the 9-valent vaccine); (3) nonvaccine subgroup (HPV 35, 39, 51, 56, 59, 68); and other HPV types.

The HPV genotypes were classified using the systems proposed by the International Agency for Research on Cancer, where the 12 HPV types 16, 18, 31, 33, 35, 39, 45, 51, 52, 56, 58, and 59 were classified as high-risk (with HPV type 68 as probably high risk) and the HPV types 26, 53, 66, 67, 70, 73, and 82 as possibly high risk.^24^

### Cervical Dysplasia

The episodes were based on the cytology examination results identified and the corresponding dates. For our analysis, we used 3 cervical disease subgroups based on cytologic diagnoses: negative for intraepithelial lesion or malignancy (NILM), low-grade precancerous lesions (including AGC-NOS, ASCUS and LSIL), high-grade precancerous lesions (including: ASC-H, HSIL, AGC-FN) and cervical cancer.

### Statistical Analysis

Age, cervical cytology grade, and HPV type-specific data are reported as frequencies and proportions for categorical variables and as mean, standard deviation (SD) and range for continuous variables (age). A specimen was counted as positive for a given HPV type if it was positive for that specific type (irrespective of potential coinfections). We assessed the hrHPV and HPV genotype–specific positivity rates by cervical disease subgroup. HPV test records within 7 days and cytology records within 30 days were considered duplicates, and only the first of the duplicate records was retained for analysis.

A specimen was considered positive for a given HPV type if it was positive for that specific type (irrespective of potential coinfections). About 75% of HPV tests were run on the extended genotyping platforms and 25% on partial genotyping platforms. In case of the latter (with pooled result), the status was categorized as high-risk-HPV-tested. These pooled aggregate test results were included in the hrHPV-positive group if the test result was positive (without type specification). In type-specific analysis, these measurements were excluded because it was not possible to extract HPV type.

We used the following case definition: woman with cervical cytology test performed and having HPV test within 9 months before or after the cervical cytology test date. This time span was based on the knowledge of the natural history (duration of high-risk) human papillomavirus infection.^25,26,27^ One woman could contribute to the case pool more than once (ie, having more than 1 cytology test performed and a matched HPV tests during the time of observation).

All cases were compiled using an open-source software tool ATLAS (developed by the OHDSI community), which facilitates the execution of analyses on standardized, patient-level, observational data in the Common Data Model (CDM) format.

Women’s ages were stratified into 4 subgroups: aged 35 years or younger, 36 to 55 years, 56 to 65 years, and 66 years or older. HPV type-specific detection percentages were determined by cervical cytology and age group with 2-sided binomial 95% CIs.

To assess the association of hrHPV infection and the grade of cervical disease, we used logistic regression to demonstrate differences between the NILM cytology group and low-grade and high-grade cervical cytology or cervical cancer with results presented as adjusted OR at 95% CI (adjusted for age). We focused on high-risk HPV types for the analyses described previously.

In estimations of the potential influence of hrHPV elimination, we assumed the direct (against HPV types targeted by vaccines) and 100% efficacy of the vaccine-induced protection. Two-sided *P* < .05 was considered statistically significant. Statistical analysis was conducted from September 2021 to August 2022 using R version 4.1.3 (R Project for Statistical Computing). SqlRender 1.7.0, DatabaseConnector 4.0.2 for database communication, and ggplot2 3.3.3 for visualizations packages were used.

## Results

### Study Population

The cohort consisted of 66 451 women aged at least 18 years (mean [SD] age, 48.1 [21.0] years). Based on the health care utilization data, over the period of observation, 40 336 women (60.7% [95% CI, 60.3%-60.7%]) had at least 1 Papanicolaou test and 7507 (11.3% [95% CI, 11.1%-11.5%]) had at least 1 HPV test.

**Figure 1.**
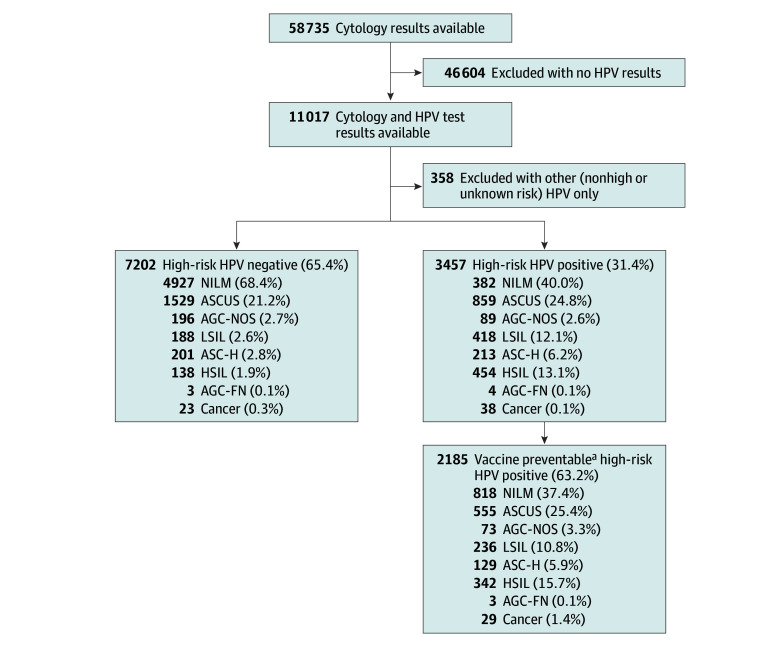
Flowchart of the Study AGC-FN indicates atypical glandular cells favor neoplasia; ASC-H, atypical squamous cells cannot exclude HSIL; AGC-NOS, atypical glandular cells not otherwise specified; ASCUS, atypical squamous cells of undetermined significance; HPV, human papillomavirus; HSIL, high-grade squamous intraepithelial lesions; LSIL, low-grade squamous intraepithelial lesions; NILM, negative for intraepithelial lesion or malignancy.

Data on the results of 58 735 Papanicolaou tests (for 30 648 women) were retrieved from the EHR. We attributed 11 017 HPV tests (for 5991 women [age range: 18 to 89 years; mean [SD] age: 39.4 [11.9] years) to a cytology test or a cervical cancer diagnosis (Table 1). Figure 1 presents the study cases and their distribution.

**Table 1.** Characteristics of Study Population and HPV Infection Detected According to Cervical Cytology Grade, Estonia, 2012 to 2019

Cervical disease subgroup	Mean (SD) [quartiles]								
	NILM cytology group	Squamous intraepithelial lesion						Cervical cancer	Total
		Low-grade			High-grade				
	NILM	ASCUS	AGC-NOS	LSIL	ASC-H	HSIL	AGC-FN	Cancer	All
No. of cases	50 397	5030	477	1189	589	852	26	175	58 735
No. of individuals	29 092	4060	420	857	524	594	26	175	30 648^a^
Age, all, y	44.2 (14.9) [32-55]	41.7 (14.4) [30-50]	45.3 (14.0) [34-54]	33.6 (11.2) [26-40]	43.3 (15.9) [30-53]	38.1 (13.4) [29-45]	54.1 (17.4) [42-70]	54.9 (15.0) [45-66]	43.7 (14.9) [32-54]
HPV testing coinciding with cervical cytology testing, No. (%)	6498 (12.9) [12.6-13.2]	2463 (49.0) [47.6-50.4]	296 (62.1) [57.5-66.4]	655 (55.1) [52.2-57.9]	421 (71.5) [67.6-75.1]	616 (72.3) [69.1-75.3]	7 (26.9) [11.6-47.8]	61 (34.9) [27.8-42.4]	11017 (18.7) [18.4-19.0]
Age of those also tested for HPV, y	39.5 (11.6) [31-47]	39.9 (11.9) [30-47]	41.9 (11.6 33-49]	34.9 (10.6 28-40]	42.7 (14.6) [31-52]	37.3 (11.8) [29-43	48.9 (16.1) [38-62]	43.2 (12.6) [34-50]	39.4 (11.9) [30-47]
Tested positive for any hrHPV	1382 (21.3) [20.3-22.3]	859 (34.5) [33-36.8]	89 (30.1) [24.9-35.6]	418 (63.8) [60.0-67.5]	213 (50.6) [45.7-55.5]	454 (73.7) [70.0-77.1]	4 (57.1) [18.4-90.1]	38 (62.3) [49.0-74.4]	3457 (31.4) [30.5-32.3]
Multiple hrHPV infection, No. (%)	288 (4.4) [3.9-5.0]	277 (11.2) [10.0-12.6]	17 (5.7) [3.4-9.0]	113 (17.3) [14.4-20.4]	43 (10.2) [7.5-13.5]	91 (14.8) [12.1-17.8]	1 (14.3) [0.4-57.9]	4 (6.6) [1.8-15.9]	834 (7.6) [7.1-8.1]

Abbreviations: AGC-FN, atypical glandular cells favor neoplasia; ASC-H, atypical squamous cells cannot exclude HSIL; AGC-NOS, atypical glandular cells not otherwise specified; ASCUS, atypical squamous cells of undetermined significance; HPV, human papillomavirus, hrHPV, high-risk HPV; HSIL, high-grade squamous intraepithelial lesions; LSIL, low-grade squamous intraepithelial lesions; NILM, negative for intraepithelial lesion or malignancy.

^a^ This is the number of unique female patients over all subgroups. One person could be in multiple subgroups over the study period.

For 73.7% (308 of 418) of LSIL, 80.0% (363 of 454) of HSIL, and 78.9% (30 of 38) of cervical cancer cases, it was possible to attribute a specific hrHPV type (the rest of the cases were considered hrHPV positive).

### Positivity Rate of hrHPV Infection

Of the women included in this analysis, the overall prevalence of hrHPV infection was 31.4% (95% CI, 30.5%-32.3%) (3457 of 11 017), ranging from the 21.3% (95% CI, 20.3%-22.3%) (1382 of 6498) among women in the NILM cytology group to 40.0% (95% CI, 38.4%-41.7%) (1366 of 3414) among women with LSIL (ASCUS, LSIL, AGC-NOS), 64.3% (95% CI, 61.3%-67.2%) (671 of 1044) among those with HSIL (ASC-H, HSIL, AGC-FN) cytology, and 62.3% (95% CI, 49.0%-74.4%) (38 of 61) among those with cervical cancer. (Table 1)

The proportions of multiple hrHPV infections detected in women was 4.4% (95% CI, 3.9%-5.0%) in the NILM cytology group, 11.9% (95% CI, 10.9%-13.1%) in the low-grade cervical cytology (ASCUS, LSIL, AGC-NOS) group, 12.9% (95% CI, 11.0%-15.1%) in the high-grade cervical cytology (ASC-H, HSIL, AGC-FN) group, and 6.5% (95% CI, 1.8%-15.9%) in the SCC group (Table 1).

High-risk HPV positivity rate was highest (18.4% [95% CI, 17.8%-19.2%]) among the youngest women (aged less than or equal to 35 years). It decreased to a very low level (less than 2%) for those aged at least 56 years (Table 2).

**Table 2.** High-risk HPV Group and Age Specific Positivity Rate by Cervical Cytology Grade, Estonia, 2012 to 2019

Cervical disease subgroup	No. (%) [95%CI]								
	NILM cytology group	Precancerous lesion						Cervical cancer	All
		Low-grade			High-grade				
	NILM^a^	ASCUS	AGC-NOS	LSIL	ASC-H	HSIL	AGC-FN	Cancer	Total
No. of cases	6498	2463	296	655	421	616	7	61	11 017
Any high-risk HPV by age group, y									
≤35	806 (12.4) [11.6-13.2]	518 (21) [19.4-22.7]	42 (14.2) [10.4-18.7]	295 (45) [41.2-48.9]	104 (24.7) [20.7-29.1]	253 (41.1) [37.2-45.1]	0	13 (21.3) [11.9-33.7]	2031 (18.4) [17.8-19.2]
36-55	458 (7) [6.4-7.7]	281 (11.4) [10.2-12.7]	37 (12.5) [9.0-16.8]	105 (16) [13.3-19.1]	68 (16.2) [12.8-20.0]	161 (26.1) [22.7-29.8]	3 (42.9) [9.9-81.6]	18 (29.5) [18.5-42.6]	1131 (10.3) [9.7-10.8]
56-65	79 (1.2) [1.0-1.5]	38 (1.5) [1.1-2.1]	5 (1.7) [0.5-3.9]	13 (2) [1.1-3.4]	19 (4.5) [2.7-7.0]	25 (4.1) [2.6-5.9]	1 (14.2) [0.4-57.9]	2 (3.3) [0.4-11.3]	182 (1.7) [1.4-1.9]
≥66	39 (0.6) [0.4-0.8]	22 (0.9) [0.6-1.3]	5 (1.7) [0.5-3.9]	5 (0.8) [0.2-1.8]	22 (5.2) [3.3-7.8]	15 (2.4) [1.4-4.0]	0	5 (8.2) [2.7-18.1]	113 (1) [0.8-1.2]
Vaccine subgroups high-risk HPV									
16, 18	420 (6.5) [5.9-7.1]	284 (11.5) [10.3-12.9]	43 (14.5) [10.7-19.1]	151 (23.1) [19.9-26.5]	93 (22.1%. 18.2-26.4]	257 (41.7) [37.8-45.7]	2 (28.6) [3.7-71.0]	24 (39.3) [27.1-52.7]	1274 (11.6) [11.0-12.2]
31, 33, 45, 52, 58	472 (7.3) [6.6-7.9]	315 (12.8) [11.5-14.2]	38 (12.8) [9.2-17.2]	117 (17.9) [15.0-21.0]	52 (12.4) [9.4-15.9]	123 (20) [16.9-23.3]	2 (28.6) [3.7-71.0]	7 (11.5) [4.7-22.2]	1126 (10.2) [9.6-10.8]
Non-vaccine hrHPV subgroups									
35, 39, 51, 56, 59, 68	558 (8.6) [7.9-9.3]	308 (12.5) [11.2-13.9]	18 (6.1) [3.6-9.4]	158 (24.1) [20.9-27.6]	49 (11.6) [8.7-15.1]	72 (11.7) [9.3-14.5]	0	2 (3.3) [0.4-11.3]	1165 (10.6) [10.0-11.2]
HPV test negative	4927 (75.8) [74.8-76.9]	1529 (62.1) [60.1-64.0]	196 (66.2) [60.5-71.6]	188 (28.7) [25.3-32.3]	201 (47.7) [42.9-52.6)	138 (22.4) [19.2-25.9]	3 (42.9) [9.9-81.6]	23 (37.7) [25.6-51.0]	7202 (65.4) [64.5-66.3]

Abbreviations: AGC-FN, atypical glandular cells favor neoplasia; ASC-H, atypical squamous cells cannot exclude HSIL; AGC-NOS, atypical glandular cells not otherwise specified; ASCUS, atypical squamous cells of undetermined significance; HPV, human papillomavirus, hrHPV, high-risk HPV; HSIL, high-grade squamous intraepithelial lesions; LSIL, low-grade squamous intraepithelial lesions; NILM, negative for intraepithelial lesion or malignancy.

^a^ Number of cases contain hrHPV positive, low-risk HPV positive, and HPV negative test results. Low-risk HPV positive tests NILM (number of cases n = 189), ASCUS (n = 75), AGC-NOS (n = 11), LSIL (n = 49), ASC-H (n = 7), HSIL (n = 24), and cancer (n = 3).

### Distribution of hrHPV Genotypes by Grade of Cervical Cytology

**Figure 2.**
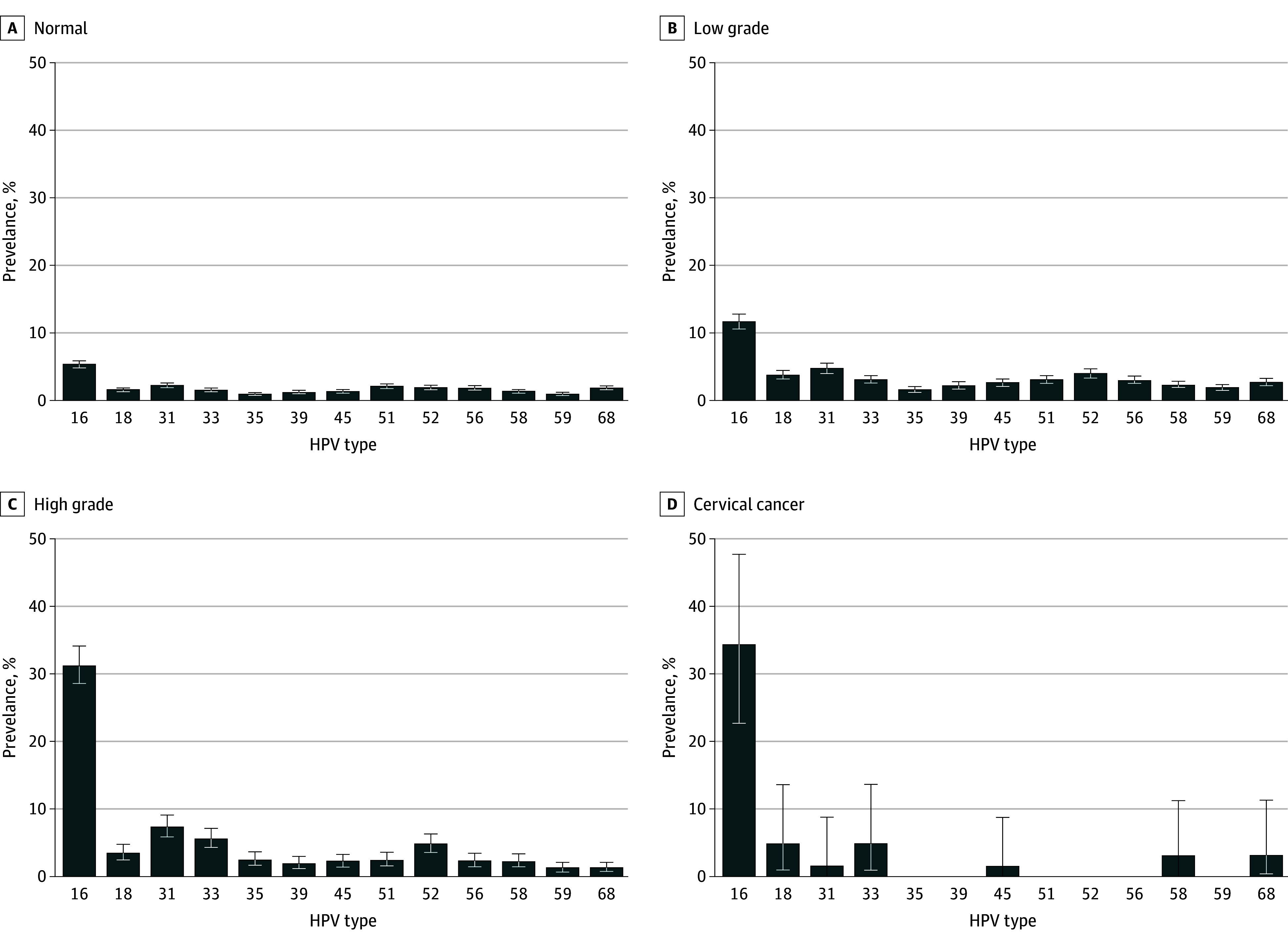
High-risk HPV Type Test Positivity by Cervical Cytology Group NILM cytology group (n = 6498), low-grade cervical cytology (ASCUS [n = 2463], LSIL [n = 655], AGC-NOS [n = 296]), high-grade cervical cytology (ASC-H [n = 421], HSIL [n = 616], AGC-FN [n = 7]), and cervical cancer (n = 61). AGC-FN indicates atypical glandular cells favor neoplasia; ASC-H, atypical squamous cells cannot exclude HSIL; AGC-NOS, atypical glandular cells not otherwise specified; ASCUS, atypical squamous cells of undetermined significance; HPV, human papillomavirus; HSIL, high-grade squamous intraepithelial lesions; LSIL, low-grade squamous intraepithelial lesions; NILM, negative for intraepithelial lesion or malignancy.

In the NILM cytology group, HPV 16 was the most common type (5.3% [95% CI, 4.8%-5.9%]), followed by HPV 31 (2.2% [95% CI, 1.8%-2.6%]), HPV 51 (2.0% [95% CI, 1.7%-2.4%]), HPV 52 (1.9% [95% CI, 1.5%-2.2%]), and HPV 56 (1.8% [95% CI, 1.5%-2.2%]) (Figure 2). (See eTable 1 in Supplement 1 for HPV type-specific positivity rate by cervical cytology grade.) In the LSIL group, HPV 16 was the most common type (11.6% [95% CI, 10.5%-12.7%]), followed by HPV 31 (4.7% [95% CI, 4.0%-5.5%]), HPV 52 (3.9% [95% CI, 3.3%-4.6%]), and HPV 18 (3.7% [95% CI, 3.1%-4.4%]).

In the HSIL group, HPV 16 prevalence was highest at 31.2% (95%CI, 28.4%-34.2%), followed by HPV 31 (7.3% [95% CI, 5.8%-9.1%]), HPV 52 (4.8% [95% CI, 3.6%-6.3%]), and HPV 33 (5.6% [95% CI, 4.3%-7.2%]). Prevalence of HPV 16 was 34.4% (95% CI, 22.8%-47.7%) in cervical cancer and 4.9% (95% CI, 1.0%-13.7%) for HPV 18 and HPV 33.

The proportion of hrHPV infections increased significantly, from the NILM to LSIL and HSIL-positive groups. Compared with hrHPV among those with NILM cervical cytology (n = 6498), the hrHPV was more common among those with LSIL (OR, 2.59 [95% CI, 2.36-2.84]), HSIL (OR, 7.14 [95% CI, 6.20-8.25]), or cervical cancer (OR, 7.28 [95% CI, 4.31-12.57]). Furthermore, the odds for the presence of 2 or more hrHPV types increased with the severity of cervical disease (in comparison with NILM, OR was 2.60 [95% CI, 2.21-3.06] for LSIL; and OR was 3.2 [95% CI 2.62-4.09] for HSIL).

### Estimated Outcomes of hrHPV Elimination (Vaccines)

HPV 16 and 18, which are covered by all HPV vaccines, were present in 36.9% (95% CI, 35.2%-38.5%) of our hrHPV-positive cytology examinations. HPV 31, 33, 45, 52, and 58, which are covered by the 9-valent vaccine, accounted for 32.6% (95% CI, 31.0%-34.2%) of HPV-positive cases in our study.

Elimination of HPV 16 and 18 were estimated to with reduce the number of women with high-grade cytology by 33.7% (95% CI, 30.9%-36.7%) and the number with low-grade cytology by 14.0% (95% CI, 12.8%-15.2%), giving an overall reduction of 18.6% (95% CI, 17.5%-19.8%) in the number of women with abnormal cytology.

Elimination of all vaccine-covered HPV types (HPV 16, 18, 31, 33, 45, 52, 58) was estimated to reduce the number of women with high-grade cytology by 50.5% (95% CI, 47.4%-53.6%) and low-grade cytology by 27.8% (95% CI, 26.3%-29.3%), giving an overall estimated reduction of 33.1% (95% CI, 31.7%-34.5%) in the number of women with abnormal cytology. The remaining hrHPV types (HPV 35, 39, 51, 56, 59, 68) were estimated to be 14.2% (95% CI, 13.0%-15.4%) of LSIL cases and 11.7% (95% CI 9.8%-13.8%) of HSIL cases.

## Discussion

This study provides insight into the occurrence of type-specific HPV and its relative contribution to the cervical precancerous disease burden among female individuals in Estonia. Surveillance of HPV infection and the associated cervical disease represents a crucial topic for monitoring and evaluating currently available antiviral prophylactic vaccines. Internationally, the etiologic fraction of HPV-associated cervical disease, based on HPV detection, varies by geography.^10,13,28^

Almost one-third (31.4%) of the female individuals tested were hrHPV-positive, with the 3 most prevalent carcinogenic types being HPV 16 (9.8%), HPV 31 (3.4%), and HPV 52 (2.8%).

Among the female individuals with normal cervical cytology, one-fifth (21.3%) tested hrHPV-positive, which is comparable to the results found among Scandinavian women.^13^ The prevalence of single high-risk HPV types was higher than reported in the meta-analysis of women with normal cytology in developed countries (for example, HPV 16: 5.3% vs 2.8%; HPV 31: 2.2% vs 1.3%; and HPV 52: 1.9% vs 1.3%).^5^ Although, in Scandinavia, the incidence of HPV 16 (4.9%), HPV 31 (2.9%), and HPV 52 (2.8%) was slightly higher. We saw that the proportion of hrHPV types increased significantly from the NILM to the LSIL and HSIL cytology groups. HrHPV was detected in 40.0% of low-grade cervical lesions and in two-thirds (64.3%) of high-grade cervical lesions. In both high-grade and low-grade cytology groups, HPV 16, 31, and 52 were detected most often.

Our finding that hrHPV prevalence increased with the severity of cervical disease reflects the well-established association between hrHPV and the risk of cervical cancer. We further found that the presence of 2 or more hrHPV types was associated with a greater likelihood of abnormal cytology, which is also consistent with previous work.^29,30^ We observed decreasing hrHPV prevalence with increasing age, consistent with previous studies,^31^ and among hrHPV-positive patients, the mean age was youngest in the LSIL group, gradually increasing in the NILM and HSIL cytology groups.

This study links nationwide databases of insurance claims and medical records, applying text mining for extraction of relevant facts. The concordance of the results with expected patterns of hrHPV distribution shows that the strategy can deliver reliable results. Such utilization of health care provision–based data provides actionable information on large and representative samples, where more rigorous sources of information are unavailable or impractical. It is estimated that of all health care data, about 80% are in an unstructured format.^32^ The complexity and volume of data in health systems means that DM and AI are to be increasingly applied to assure use of data for disease prevention, creating early diagnosis and screening programs, monitoring treatment response, health care planning, and decision making. An alternative (ie, not using DM and AI) would be just creating digital waste and administrative costs.

### Limitations and Strengths

Our findings must be considered within the limitations of the data and study design. Using routinely collected data for clinical research has challenges.^33,34^ As we identified both cervical disease states and cervical HPV testing episodes from the EHR collection through the identification and exploration of a considerable volume of unstructured or semistructured data, we had to consider inaccuracies in the data on HPV and cytology results. First, there may be completeness issues due to missing results not being sent to the central repository and not all analysis results being present. Thus, underestimation of the number of cytology cases and HPV testing might occur. Second, there may be consistency problems, where 1 measurement appears in multiple documents with slightly different results or different dates (eg, date on which the specimen was taken vs date on which the analysis was performed or recorded). Third, both clinical tests can be applied in contexts unrelated to cervical cancer prevention and diagnostics. To manage all of the aforementioned challenges, we used rigorous criteria for case definitions and linking of the events, using clinical context from different sources and merging temporally close results, according to the clinical knowledge of the disease progression and treatment. Also, despite the large number of female individuals in the overall sample, the number of cervical cytology results indicative of cervical cancer with a linked HPV test result was too low for meaningful analysis. Additionally, relying on aggregated HPV assays led to underestimating the prevalence of pooled hrHPV types (31/33/35/39/45/51/52/56/58/59/66/68) by approximately 20%. However, estimates of HPV 16, HPV 18, and the total hrHPV prevalence are not affected by this measurement bias.

Still, this study has notable strengths. This study describes HPV type distribution by cervical cytology grade in the largest known sample from the European region with the highest incidence and mortality from cervical cancer.^35^ As Estonian health record system integrates data from all health care professionals (hospitals, family physicians, laboratories, and others) for all patients^21^, the data lacks systematic biases inherent in many other data sources. Therefore, the results are representative and generalizable in Estonian context.

## Conclusions

In this cross-sectional study, text mining and natural language processing techniques allowed the detection of precursors to cervical cancer based on data stored by the nationwide health system. Our findings contribute to the literature on type-specific HPV distribution by cervical cytology grade and document that α-9 phylogenetic group HPV types 16, 31, 33, 52, and α-7 phylogenetic group HPV 18 are the most frequently detected in normal to high-grade precancerous lesions in Estonia.

## Notes

Supplement 1. **eTable 1.** HPV Testing Coinciding With Cervical Cytology Testing by Age Group, and the Mean Age of Those Tested hrHPV Positive, Estonia 2012-2019 **eTable 2.** HPV Type-Specific Positivity Rate of by Cervical Cytology Grade, Estonia 2012-2019 **eTable 3.** Concept Sets Used

Supplement 2.
Data Sharing Statement
