# Supplementary material for: Prevaccination Prevalence of Type-Specific Human Papillomavirus Infection by Grade of Cervical Cytology in Estonia
Source: JAMA Netw Open. 2023 Feb 6;6(2):e2254075. doi: 10.1001/jamanetworkopen.2022.54075 (PMC12527416; doi:10.1001/jamanetworkopen.2022.54075)
Supplement: Supplement 1. — eTable 1. HPV Testing Coinciding With Cervical Cytology Testing by Age Group, and the Mean Age of Those Tested hrHPV Positive, Estonia 2012-2019 eTable 2. HPV Type-Specific Positivity Rate of by Cervical Cytology Grade, Estonia 2012-2019 eTable 3. Concept Sets Used [file jamanetwopen-e2254075-s001.pdf]

Supplemental Online Content

Uusküla A, Oja M, Tamm S, et al. Prevaccination prevalence of type-specific human papillomavirus infection by grade of cervical cytology in Estonia. *JAMA Netw Open.* 2023;6(2):e2254075. doi:10.1001/jamanetworkopen.2022.54075

eTable 1. HPV Testing Coinciding With Cervical Cytology Testing by Age Group, and the Mean Age of Those Tested hrHPV Positive, Estonia 2012-2019

eTable 2. HPV Type-Specific Positivity Rate of by Cervical Cytology Grade, Estonia 2012-2019

eTable 3. Concept Sets Used

This supplemental material has been provided by the authors to give readers additional information about their work.

© 2023 Uusküla A et al. *JAMA Network Open.*

eTable 1. HPV testing coinciding with cervical cytology testing by age group, and the mean age of those tested hrHPV positive, Estonia 2012-2019

Cervical disease subgroup	NILM cytology group [n (%, 95%CI)]	Low-grade squamous intraepithelial lesion [n (%, 95%CI)]			High-grade squamous intraepithelial lesion [n (%, 95%CI)]			Cervical cancer [n (%, 95%CI)]	Total [n (%, 95% CI)]
Aged ≤35 years	2828 (43.5%, 42.3-44.7%)	1059 (43.0%, 41.0-45.0%)	102 (34.4%, 29.1-40.2%)	418 (63.8%, 60.0-67.5%)	154 (36.6%, 32.0-41.4%)	334 (54.2%, 50.2-58.2%)	3 (42.9%, 9.9-81.6%)	19 (31.1%, 19.9-44.3%)	4917 (44.6%, 43.7-45.6%)
Aged 36-55 years	3016 (46.4%, 45.2-47.6%)	1159 (47.1%, 45.1-49.1%)	158 (53.4%, 47.5-59.2%)	203 (31.0%, 27.5-34.7%)	192 (45.6%, 40.8-50.5%)	224 (36.4%, 32.6-40.3%)	1 (14.3%, 0.4-57.9%)	34 (55.7%, 42.4-68.5%)	4987 (45.3%, 44.3-46.2%)
Aged 56-65 years	477 (7.3%, 6.7-8.8%)	145 (5.9%, 5.0-6.9%)	23 (7.8%, 5.0-11.4%)	23 (3.5%, 2.2-5.2%)	39 (9.3%, 6.7-12.4%)	39 (6.3%, 4.5-8.6%)	1 (14.3%, 0.4-57.9%)	2 (3.3%, 0.4-11.3%)	749 (6.8%, 6.3-7.3%)
Aged ≥66 years	177 (2.8%, 2.3-3.1%)	100 (4.1%, 3.3-4.9%)	13 (4.4%, 2.4-7.4%)	11 (1.7%, 0.8-3.0%)	36 (8.6%, 6.1-11.6%)	19 (3.1%, 1.9-4.8%)	2 (28.6%, 3.7-71.0%)	6 (9.8%, 3.7-20.2%	364 (3.3%, 3.0-3.7%)
Age (years) of those tested hrHPV positive (mean, (SD, quartiles))	36.5 (12.0, 28-43)	36.1 (11.4, 28-42)	39.5 (12.0, 31-45)	33.4 (10.0 27-38)	40.4 (15.8, 28-49)	36.7 (11.7, 28-41)	44.3 (9.1, 39-48)	43.2 (13.9, 33-52)	36.4 (12.0, 28-43)

© 2023 Uusküla A et al. *JAMA Network Open.*

eTable 2. HPV type-specific positivity rate of by cervical cytology grade, Estonia 2012-2019

**Cervical disease subgroup**	**NILM cytology group (n)**	**Low-grade precancerous lesion (n)**			**High-grade precancerous lesion (n)**			**Cervical cancer (n)**	**All (N)**
	NILM	ASCUS	AGC-NOS	LSIL	ASC-H	HSIL	AGC-FN	Cancer	Total
Number of cases	6498	2463	296	655	421	616	7	61	11017
**High-risk HPV types [n (%)]**									
HPV type 16	344 (5.3%)	234 (9.5%)	36 (12.2%)	125 (19.1%)	82 (19.5%)	240 (39%)	2 (28.6%)	21 (34.4%)	1084 (9.8%)
HPV type 18	96 (1.5%)	77 (3.1%)	8 (2.7%)	42 (6.4%)	13 (3.1%)	23 (3.7%)		3 (4.9%)	262 (2.4%)
HPV type 31	142 (2.2%)	103 (4.2%)	14 (4.7%)	44 (6.7%)	20 (4.8%)	55 (8.9%)	1 (14.3%)	1 (1.6%)	380 (3.4%)
HPV type 33	97 (1.5%)	73 (3%)	8 (2.7%)	22 (3.4%)	16 (3.8%)	42 (6.8%)		3 (4.9%)	261 (2.4%)
HPV type 35	58 (0.9%)	34 (1.4%)	1 (0.3%)	18 (2.7%)	6 (1.4%)	20 (3.2%)		0 (0%)	137 (1.2%)
HPV type 39	76 (1.2%)	47 (1.9%)	1 (0.3%)	25 (3.8%)	9 (2.1%)	11 (1.8%)		0 (0%)	169 (1.5%)
HPV type 45	84 (1.3%)	64 (2.6%)	5 (1.7%)	19 (2.9%)	10 (2.4%)	13 (2.1%)		1 (1.6%)	196 (1.8%)
HPV type 51	133 (2%)	61 (2.5%)	3 (1%)	39 (6%)	16 (3.8%)	9 (1.5%)		0 (0%)	261 (2.4%)
HPV type 52	121 (1.9%)	81 (3.3%)	11 (3.7%)	42 (6.4%)	12 (2.9%)	37 (6%)	1 (14.3%)	0 (0%)	305 (2.8%)
HPV type 56	117 (1.8%)	51 (2.1%)	5 (1.7%)	45 (6.9%)	10 (2.4%)	14 (2.3%)		0 (0%)	242 (2.2%)
HPV type 58	84 (1.3%)	46 (1.9%)	9 (3%)	23 (3.5%)	7 (1.7%)	16 (2.6%)		2 (3.3%)	187 (1.7%)
HPV type 59	58 (0.9%)	45 (1.8%)	2 (0.7%)	16 (2.4%)	5 (1.2%)	8 (1.3%)		0 (0%)	134 (1.2%)
HPV type 68	116 (1.8%)	70 (2.8%)	6 (2%)	15 (2.3%)	3 (0.7%)	10 (1.6%)		2 (3.3%)	222 (2%)
Any high-risk HPV positive	1382 (21.3%)	859 (34.9%)	89 (30.1%)	418 (63.8%)	213 (50.6%)	454 (73.7%)	4 (57.1%)	38 (62.3%)	3453 (31.4%)
**Low-risk HPV types [n (%)]**									
HPV type 11	6 (0.1%)	2 (0.1%)	0 (0%)	2 (0.3%)	0 (0%)	3 (0.5%)		0 (0%)	13 (0.1%)
HPV type 40	6 (0.1%)	7 (0.3%)	3 (1%)	2 (0.3%)	1 (0.2%)	7 (1.1%)		0 (0%)	26 (0.2%)
HPV type 42	22 (0.3%)	3 (0.1%)	0 (0%)	3 (0.5%)	0 (0%)	6 (1%)		0 (0%)	34 (0.3%)
HPV type 43	13 (0.2%)	5 (0.2%)	0 (0%)	3 (0.5%)	0 (0%)	3 (0.5%)		0 (0%)	24 (0.2%)
HPV type 44	9 (0.1%)	5 (0.2%)	2 (0.7%)	4 (0.6%)	1 (0.2%)	2 (0.3%)		0 (0%)	23 (0.2%)
HPV type 53	79 (1.2%)	41 (1.7%)	6 (2%)	14 (2.1%)	9 (2.1%)	14 (2.3%)		1 (1.6%)	164 (1.5%)
HPV type 54	23 (0.4%)	1 (0%)	0 (0%)	1 (0.2%)	0 (0%)	5 (0.8%)		0 (0%)	30 (0.3%)
HPV type 61	14 (0.2%)	9 (0.4%)	3 (1.0%)	2 (0.3%)	0 (0%)	6 (1.0%)		0 (0%)	34 (0.3%)
HPV type 66	110 (1.7%)	62 (2.5%)	1 (0.3%)	33 (5.0%)	7 (1.7%)	8 (1.3%)		1 (1.6%)	222 (2%)
HPV type 70	10 (0.2%)	1 (0%)	0 (0%)	0 (0%)	0 (0%)	0 (0%)		0 (0%)	11 (0.1%)

© 2023 Uusküla A et al. *JAMA Network Open.*

HPV type 73	13 (0.2%)	5 (0.2%)	0 (0%)	2 (0.3%)	0 (0%)	4 (0.6%)		0 (0%)	24 (0.2%)
HPV type 82	29 (0.4%)	19 (0.8%)	4 (1.4%)	9 (1.4%)	2 (0.5%)	2 (0.3%)		1 (1.6%)	65 (0.6%)

*HPV 68 – probably high-risk HPV type

eTable 3. Concept Sets Used

1.1 HPV test from measurements ([CCS] hpv_test)

Concept id	Code	Name	Vocabulary
42870371	71432-9	Human papilloma virus 16 and 18 and 31+33+35+39+45+51+52+56+58+59+66+68 DNA - Cervix	LOINC
42870370	71431-1	Human papilloma virus 31+33+35+39+45+51+52+56+58+59+66+68 DNA [Presence] in Cervix by NAA with probe detection	LOINC
42868547	70061-7	Human papilloma virus 16 and 18 DNA [Presence] in Specimen by NAA with probe detection	LOINC
40764134	61373-7	Human papilloma virus 18 DNA [Presence] in Specimen by NAA with probe detection	LOINC
40764133	61372-9	Human papilloma virus 16 DNA [Presence] in Specimen by NAA with probe detection	LOINC
4262119	35904009	Human papillomavirus DNA detection	SNOMED
3033110	42770-8	Human papilloma virus high and Low risk DNA panel - Cervix	LOINC
3032431	49896-4	Human papilloma virus 16+18+31+33+35+39+45+51+52+56+58+59+68 DNA [Presence] in Specimen by NAA with probe detection	LOINC

1.2 HPV test positive from conditions ([CCS] hpv_test_positive_from_conditions)

Concept id	Code	Name	Vocabulary
40480043	441667007	Abnormal cervical Papanicolaou smear with positive human papillomavirus deoxyribonucleic acid test	SNOMED
37206940	787723002	Human papillomavirus deoxyribonucleic acid test positive	SNOMED
37109025	16079011000119105	Human papillomavirus deoxyribonucleic acid test positive, low risk on cervical specimen	SNOMED
36717114	720005005	Human papillomavirus deoxyribonucleic acid test positive, high risk on cervical specimen	SNOMED

1.3 HPV test high risk ([CCS] hpv_test_high_risk)

Concept id	Code	Name	Vocabulary
42870371	71432-9	Human papilloma virus 16 and 18 and 31+33+35+39+45+51+52+56+58+59+66+68 DNA - Cervix	LOINC
42870370	71431-1	Human papilloma virus 31+33+35+39+45+51+52+56+58+59+66+68 DNA [Presence] in Cervix by NAA with probe detection	LOINC

© 2023 Uusküla A et al. *JAMA Network Open.*

42868547	70061-7	Human papilloma virus 16 and 18 DNA [Presence] in Specimen by NAA with probe detection	LOINC
40764134	61373-7	Human papilloma virus 18 DNA [Presence] in Specimen by NAA with probe detection	LOINC
40764133	61372-9	Human papilloma virus 16 DNA [Presence] in Specimen by NAA with probe detection	LOINC
3032431	49896-4	Human papilloma virus 16+18+31+33+35+39+45+51+52+56+58+59+68 DNA [Presence] in Specimen by NAA with probe detection	LOINC

1.4 HPV test positive - high risk from conditions ([CCS] hpv_test_high_risk_positive_from_conditions)

Concept Id	Code	Name	Vocabulary
36717114	720005005	Human papillomavirus deoxyribonucleic acid test positive, high risk on cervical specimen	SNOMED

1.5 HPV test for types 16/18 ([CCS] hpv_test_16/18)

Concept id	Code	Name	Vocabulary
42868547	70061-7	Human papilloma virus 16 and 18 DNA [Presence] in Specimen by NAA with probe detection	LOINC
40764134	61373-7	Human papilloma virus 18 DNA [Presence] in Specimen by NAA with probe detection	LOINC
40764133	61372-9	Human papilloma virus 16 DNA [Presence] in Specimen by NAA with probe detection	LOINC

1.6 HPV negative test from conditions ([CCS] hpv_test_negative_from_conditions)

Concept id	Code	Name	Vocabulary
37206941	787724008	Human papillomavirus deoxyribonucleic acid test negative	SNOMED

1.7 [CCS] ASCUS

Concept id	Code	Name	Vocabulary
4191603	39035006	Atypical squamous cells of undetermined significance	SNOMED

1.8 [CCS] NILM

Concept id	Code	Name	Vocabulary
4162714	373887005	Negative for intraepithelial lesion or malignancy	SNOMED

1.9 [CCS] AGC-NOS

Concept id	Code	Name	Vocabulary

© 2023 Uusküla A et al. *JAMA Network Open.*

4304347	103646000	Atypical endometrial cells of undetermined significance	SNOMED
4304331	103643008	Atypical endocervical cells of undetermined significance	SNOMED
4260390	4476003	Atypical glandular cells of undetermined significance	SNOMED

1.10 [CCS] LSIL

Concept id	Code	Name	Vocabulary
4013220	112662005	Low-grade squamous intraepithelial lesion	SNOMED

1.11 [CCS] ASC-h

Concept id	Code	Name	Vocabulary
4161591	373878001	Atypical squamous cells, cannot exclude HSIL	SNOMED

1.12 [CCS] HSIL

Concept id	Code	Name	Vocabulary
4331440	22725004	High-grade squamous intraepithelial lesion	SNOMED

1.13 [CCS] AGC-FN

Concept id	Code	Name	Vocabulary
4162712	373883009	Atypical glandular cells, favor neoplastic	SNOMED
4162711	373882004	Atypical endocervical cells, favor neoplastic	SNOMED

1.14 [CCS] AIS

Concept id	Code	Name	Vocabulary
40486538	447760009	Endocervical adenocarcinoma in situ	SNOMED
4112875	254890008	Adenocarcinoma in situ of cervix	SNOMED

1.15 [CCS] pap_test

Concept id	Code	Name	Vocabulary
43531329	609040007	Microscopic cytologic examination of smear of specimen from female genital tract prepared using Papanicolaou technique	SNOMED
4331440	22725004	High-grade squamous intraepithelial lesion	SNOMED
4304347	103646000	Atypical endometrial cells of undetermined significance	SNOMED
4304331	103643008	Atypical endocervical cells of undetermined significance	SNOMED
4260390	4476003	Atypical glandular cells of undetermined significance	SNOMED

© 2023 Uusküla A et al. *JAMA Network Open.*

4208622	440623000	Microscopic examination of cervical Papanicolaou smear	SNOMED
4191603	39035006	Atypical squamous cells of undetermined significance	SNOMED
4162714	373887005	Negative for intraepithelial lesion or malignancy	SNOMED
4162712	373883009	Atypical glandular cells, favor neoplastic	SNOMED
4161591	373878001	Atypical squamous cells, cannot exclude HSIL	SNOMED
4112875	254890008	Adenocarcinoma in situ of cervix	SNOMED
4013220	112662005	Low-grade squamous intraepithelial lesion	SNOMED
3025156	19765-7	Microscopic observation [Identifier] in Cervical or vaginal smear or scraping by Cyto stain	LOINC
765403	448651000124104	Microscopic examination of cervical Papanicolaou smear and Human papillomavirus deoxyribonucleic acid detection cotesting	SNOMED
433033	439888000	Abnormal cervical Papanicolaou smear	SNOMED

1.16 [CCS] malignant_neoplasm_of_cervix

Concept id	Code	Name	Vocabulary
4247367	406103009	Squamous cell carcinoma in situ of uterine cervix	SNOMED
4243874	92588008	Carcinoma in situ of exocervix	SNOMED
4243120	92580001	Carcinoma in situ of endocervix	SNOMED
4116080	254886006	Squamous cell carcinoma of cervix	SNOMED
4112875	254890008	Adenocarcinoma in situ of cervix	SNOMED
4110872	254887002	Adenocarcinoma of cervix	SNOMED
4092515	188180002	Malignant neoplasm, overlapping lesion of cervix uteri	SNOMED
441805	93779009	Primary malignant neoplasm of endocervix	SNOMED
436358	93789008	Primary malignant neoplasm of exocervix	SNOMED
198984	363354003	Malignant tumor of cervix	SNOMED
196359	372024009	Primary malignant neoplasm of uterine cervix	SNOMED
194611	92564006	Carcinoma in situ of uterine cervix	SNOMED

© 2023 Uusküla A et al. *JAMA Network Open.*
