# Supplementary material for: Prevaccination Prevalence of Type-Specific Human Papillomavirus Infection by Grade of Cervical Cytology in Estonia
Source: JAMA Netw Open. 2023 Feb 6;6(2):e2254075. doi: 10.1001/jamanetworkopen.2022.54075 (PMC12527416; doi:10.1001/jamanetworkopen.2022.54075)
Supplement: Supplement 2. — Data Sharing Statement [file jamanetwopen-e2254075-s002.pdf]

Data Sharing Statement

Uusküla. Prevaccination Prevalence of Type-Specific Human Papillomavirus Infection by Grade of Cervical Cytology in Estonia. *JAMA Netw Open*. Published February 06, 2023. doi:10.1001/jamanetworkopen.2022.54075

Data
Data available: No

Additional Information
Explanation for why data not available: There are legal restrictions on sharing deidentified data received from health data registers in Estonia. The data can be requested by completing an application to The Health and Welfare Information Systems Center (TEHIK) (https://www.tehik.ee/en/statistics.
